# Comparative Evaluation of Tongue Crib and Tongue Tamers in Repositioning the Tongue in Subjects With Tongue Thrusting: A Randomized Clinical Trial

**DOI:** 10.7759/cureus.113646

**Published:** 2026-07-30

**Authors:** Simmi Arora, Pradeep Raghav, Ruchi Saini, Anamika Sharma, Manvi Agarwal

**Affiliations:** 1 Department of Orthodontics and Dentofacial Orthopedics, Subharti Dental College and Hospital, Meerut, IND

**Keywords:** cone beam computed tomography, habit breaking appliance, tongue crib, tongue tamers, tongue thrusting habit, visual analog scale

## Abstract

Background

Tongue thrusting is a common neuromuscular habit associated with abnormal tongue posture and dentoalveolar changes. Tongue crib and tongue tamers are commonly used habit-breaking appliances, but limited studies have compared their effectiveness using cone beam computed tomography (CBCT) analysis.

Aim

This study aims to compare the efficacy of tongue crib and tongue tamers in repositioning the tongue in patients with tongue thrust using CBCT analysis.

Materials and methods

Forty subjects aged 14-30 years with tongue thrusting habit were randomly divided into two groups: tongue crib (Group 1) and tongue tamers (Group 2). CBCT with barium sulphate coating was used to evaluate tongue posture. Parameters assessed included tongue tip position, U1-PP, L1-MP, interincisal angle, overjet, overbite, and lower lip retraction. Patient discomfort was evaluated using a visual analog scale (VAS). Statistical analysis was performed with significance set at p ≤ 0.05.

Results

Tongue tip position and dentoalveolar parameters significantly improved with both appliances. Compared to tongue tamers, tongue crib showed statistically significant improvement in tongue tip retraction and overbite improvement. On the other hand, tongue tamers demonstrated greater patient acceptability and comfort.

Conclusion

Both tongue crib and tongue tamers were effective in repositioning the tongue and improving associated dentoalveolar changes, but the tongue crib was more effective clinically, whereas tongue tamers provided better patient comfort.

## Introduction

The tongue plays an essential role in speech, swallowing, mastication, and craniofacial growth [1]. Equilibrium between the tongue, lips, and cheeks is necessary for the maintenance of normal occlusion and dental arch stability. Altered tongue posture or abnormal tongue function may disturb this equilibrium, leading to dentofacial abnormalities and malocclusions [2]. Tongue thrusting is characterized by the forward placement of the tongue against or between the teeth during swallowing, speech, and sometimes at rest. Simple tongue thrust is usually associated with an anterior open bite and normal posterior occlusion, in which the posterior teeth come into contact during swallowing, and the condition remains primarily dental in nature with minimal muscular imbalance. In contrast, complex tongue thrust is characterized by a diffuse open bite, a lack of proper posterior occlusal contact during swallowing, and excessive perioral muscle activity. It is often associated with mouth breathing, enlarged tonsils or adenoids, chronic naso-respiratory disorders, and skeletal discrepancies [3].

Various etiological factors have been implicated in tongue thrusting, including hereditary influences, prolonged thumb sucking, mouth breathing, chronic tonsillitis, upper respiratory tract infections, and improper bottle feeding [4,5]. The prevalence of tongue thrusting decreases with age but remains a common finding among orthodontic patients [6].

Management of tongue thrusting includes myofunctional therapy [7] and habit-breaking appliances such as tongue crib, tongue beads, bonded spurs, and tongue tamers [8]. The tongue crib acts as a mechanical barrier preventing anterior tongue positioning, whereas tongue tamers function through proprioceptive feedback and neuromuscular re-education [9]. Despite their clinical effectiveness, both tongue crib and tongue tamers are associated with certain functional limitations, including difficulties in speech and eating, tongue irritation, general discomfort, and esthetic concerns. As these appliances occupy intraoral space, they may interfere with normal oral functions and are often not well tolerated by patients [10].

Most previous studies have evaluated dentoalveolar changes associated with these appliances using lateral cephalograms. However, conventional radiographs fail to provide a three-dimensional (3D) assessment of tongue posture due to the superimposition of structures. Cone beam computed tomography (CBCT) offers accurate 3D evaluation of tongue morphology and position with relatively low radiation exposure and upright patient positioning [11].

Limited literature exists comparing tongue crib and tongue tamers in repositioning the tongue using CBCT analysis. Therefore, the present randomized clinical trial was conducted to evaluate and compare the efficacy of tongue crib and tongue tamers in repositioning the tongue in tongue thrusting subjects using CBCT analysis.

## Materials and methods

Ethical approval of this single-center randomized clinical trial was obtained from the research ethics committee of the Swami Vivekanand Subharti University, Meerut. This study was registered at the Clinical Trial Registry India (CTRI/2026/01/100132). An explanation about the study was given to the patients. Those agreeing to participate completed a written consent form.

Sample size calculation

The smallest required sample size was calculated to be 20 participants, with 10 participants in each group, based on the population proportion and population size of Western Uttar Pradesh, keeping a 95% confidence interval and a 5% margin of error. The sample size was calculated using the formula for comparison of two independent means with the help of OpenEpi 3.0 software (Dean AG, Sullivan KM, Soe MM, Emory University, Atlanta, GA):

$n_{1} = \frac{ \left(\dfrac{\sigma_{1}^{2}+\sigma_{2}^{2}}{k}\right) \left(z_{1-\alpha/2}+z_{1-\beta}\right)^{2} }{ D^{2} }$

$n_{2}=\frac{(k^{*}\sigma_{1}^{2}+\sigma_{2}^{2})(z_{1-\alpha/2}+z_{1-\beta})^{2}}{D^{2}}$

where n is the sample size per group, σ is the standard deviation, d is the minimum clinically significant difference between groups, k is the ratio n2/n1, Z_1-α/2_ corresponds to the selected significance level (α = 0.05), and Z_1-β_ corresponds to the desired study power (90%).

**Figure 1:**
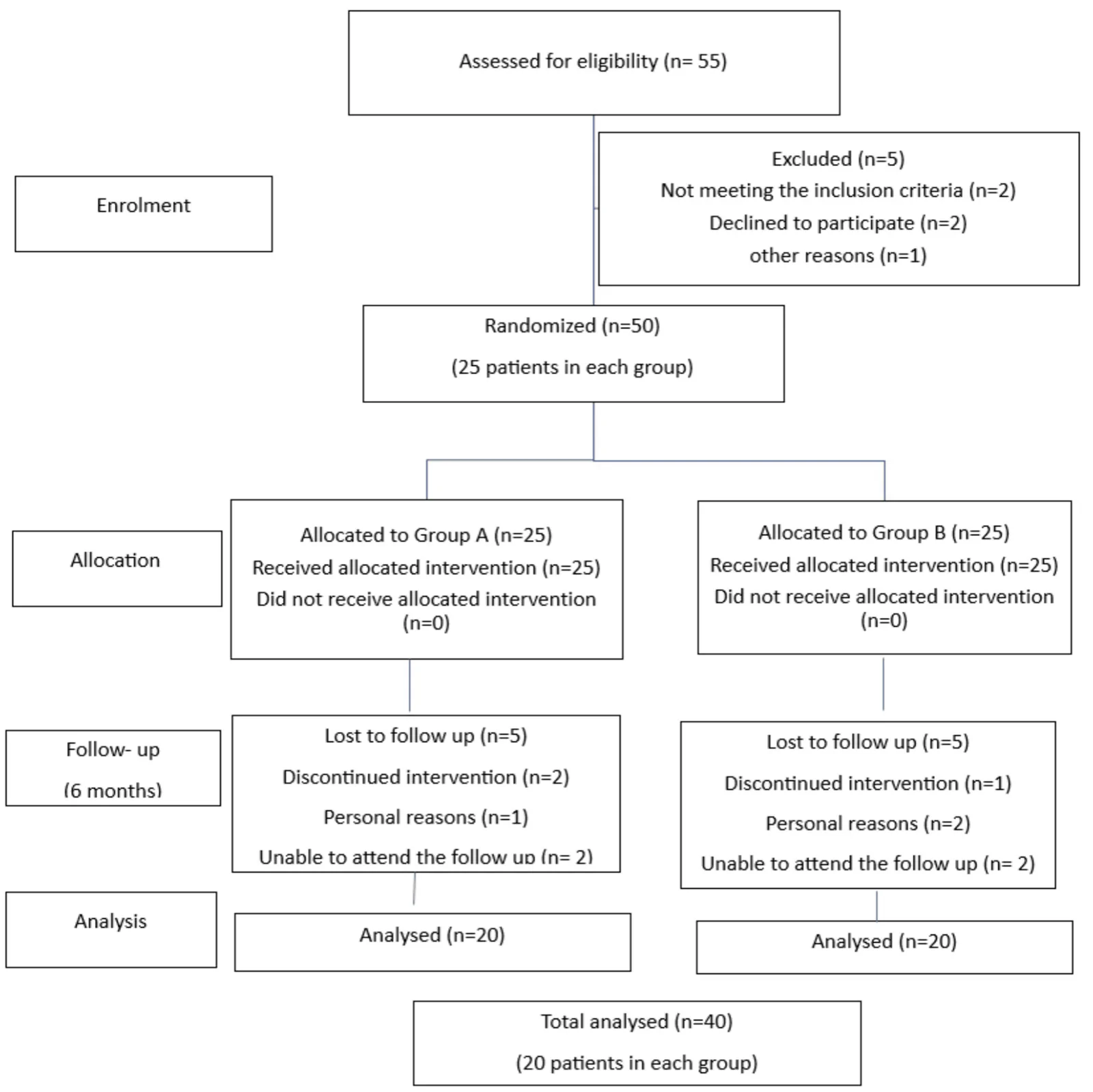
CONSORT flow diagram

To increase the reliability and clinical relevance of the study, the sample size was doubled, resulting in 40 participants, with 20 participants allocated to each group. Also, considering a potential attrition rate during the follow-up period, 25 patients were initially enrolled in each group. During the six-month follow-up period, some subjects were lost to follow-up, resulting in a final sample size of 20 participants in each group (Figure 1).

Eligibility criteria

Patients were enrolled in the study if they met the following inclusion criteria: subjects between 14 and 30 years of age, having a simple tongue thrusting habit with proclined/spaced upper anterior teeth, proclined/retroclined lower incisors, having difficulty in chewing and speaking, and a convex profile. Subjects were excluded if they had crowding with respect to the upper/lower teeth, diffuse open bite, loss of permanent anterior teeth, tooth agenesis, systemic or endocrinological disorder, craniofacial anomalies, neuromuscular disorder affecting tongue position, trauma and surgery involving the tongue, and a history of orthodontic treatment.

Subject recruitment

Forty patients who met the inclusion and exclusion criteria were randomly allocated into two groups. To minimize selection bias, simple randomization was carried out with a 1:1 allocation ratio. Treatment allocation was concealed using sequentially numbered, sealed, opaque envelopes that were shuffled by an independent investigator. Each patient selected one envelope to determine their group. The investigator was blinded to the randomization process. Participants were divided into two groups: Group 1 received the tongue crib appliance, and Group 2 received the tongue tamers appliance.

Examination of tongue thrust swallow

Tongue thrust swallowing can only be examined clinically. In order to examine the presence of tongue thrusting, the subjects were asked to swallow their saliva three times during the same visit. To enhance the reliability of the examination, the clinical evaluation of each subject was done by two independent clinicians. A final diagnosis was made only after both clinicians reached agreement on the presence of tongue thrusting.

Interventions

**Figure 2:**
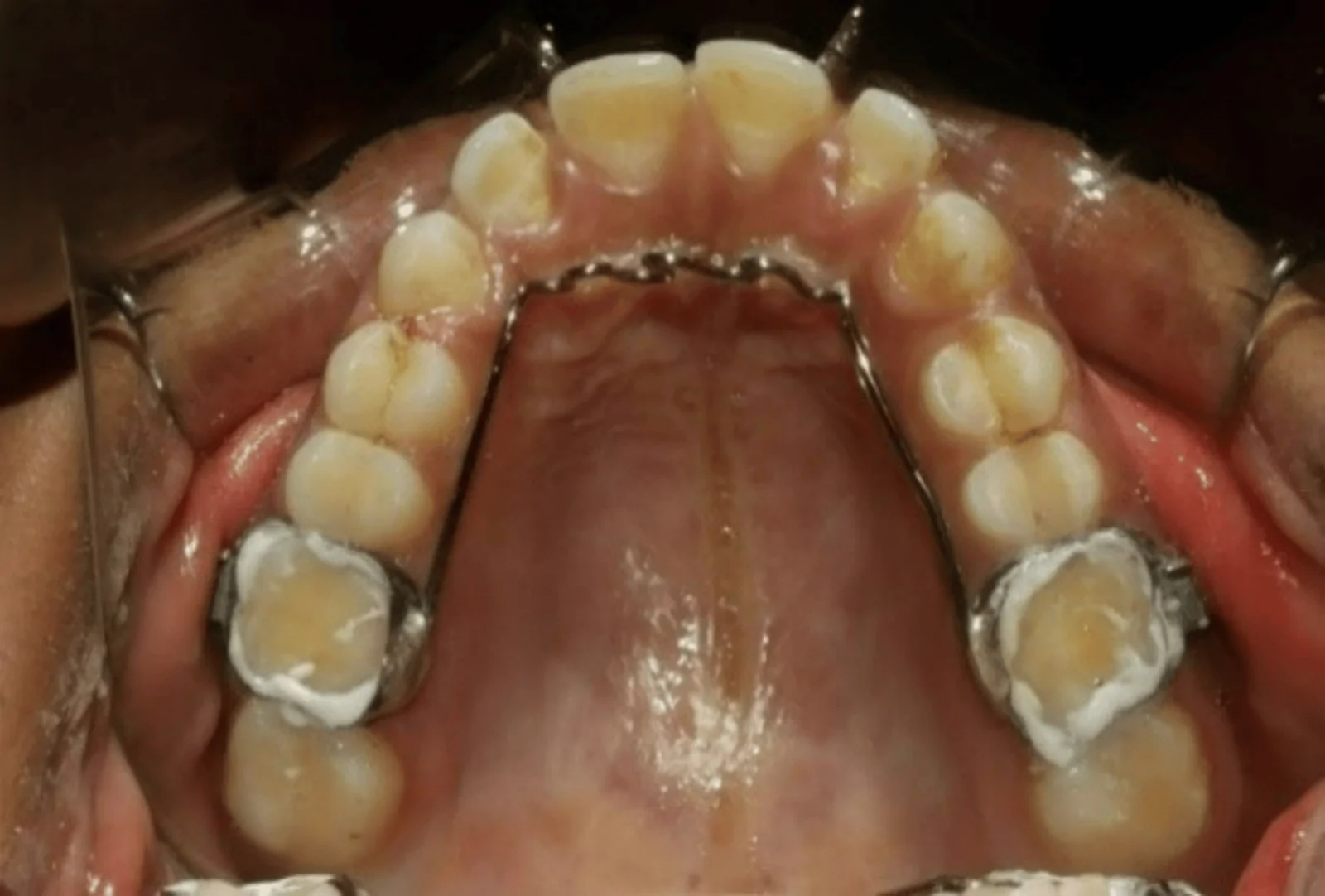
Tongue crib This figure shows the tongue crib appliance fabricated using 21-gauge stainless steel wire. Vertical cribs measuring 6-10 mm were positioned on the anterior palatal aspect of maxillary anterior teeth.

The tongue crib appliance was fabricated using 21-gauge stainless steel wire soldered to orthodontic bands extending between the maxillary canines. Vertical cribs measuring 6-10 mm were positioned on the anterior palatal aspect of the maxillary anterior teeth and cemented using glass ionomer cement (Figure 2).

**Figure 3:**
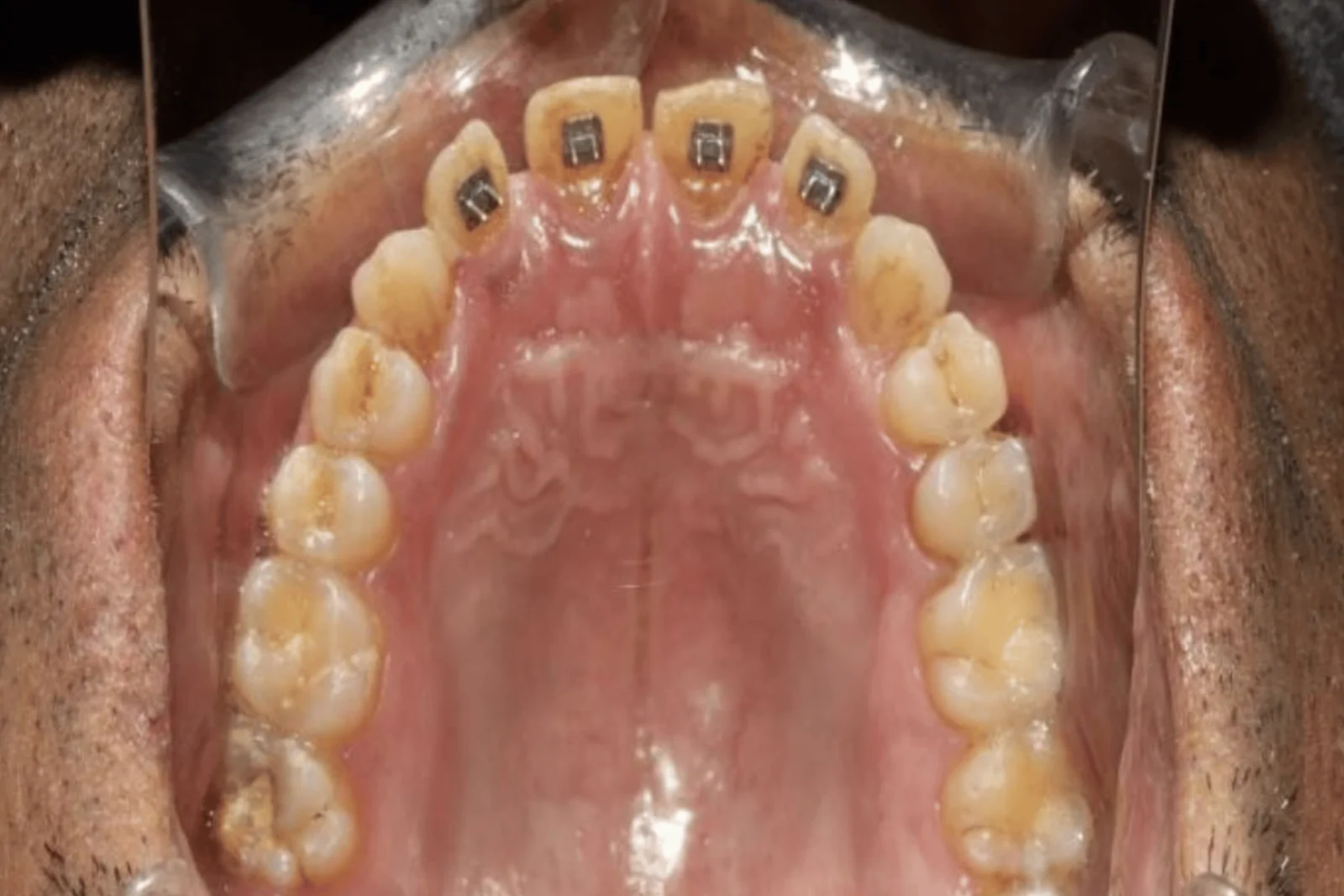
Tongue tamers This figure shows tongue tamers bonded on to the palatal aspect of maxillary central and lateral incisors.

Tongue tamers (Genius Ortho Pvt. Ltd., Rajkot, Gujarat, India) were bonded depending on the position of the tongue onto the palatal surfaces of maxillary incisors or lingual surfaces of mandibular incisors using Transbond XT composite resin after acid etching and priming (Figure 3) [12].

Both the tongue crib and tongue tamers appliances were delivered for a treatment duration of six months. Follow-up appointments were scheduled at biweekly intervals during the first month and monthly thereafter to evaluate appliance performance, monitor treatment progress, and ensure patient compliance with appliance wear. 

Barium sulphate suspension was prepared by centrifuging 1 g of barium sulphate at 4000 rpm for 10 minutes. The prepared suspension was applied over the dorsal surface of the tongue to enhance visualization of the soft tissue boundaries, as it acts as a radiopaque contrast agent.

Imaging technique

CBCT scans were obtained for all subjects in both the tongue crib and tongue tamers groups before treatment initiation and after six months of appliance therapy. Images were acquired using the Sirona Galileos CBCT software (Dentsply Sirona, Charlotte, NC) with an effective radiation dose ranging from 15-273 µSv and an isotropic voxel size of 0.16 mm. The field of view was 11 × 11 cm, with imaging parameters of 85 kV, 6 mA, and a scan time of 14.2 seconds. Subjects were positioned in a natural head posture with the Frankfort horizontal plane parallel to the floor during image acquisition.

Outcome measures

**Figure 4:**
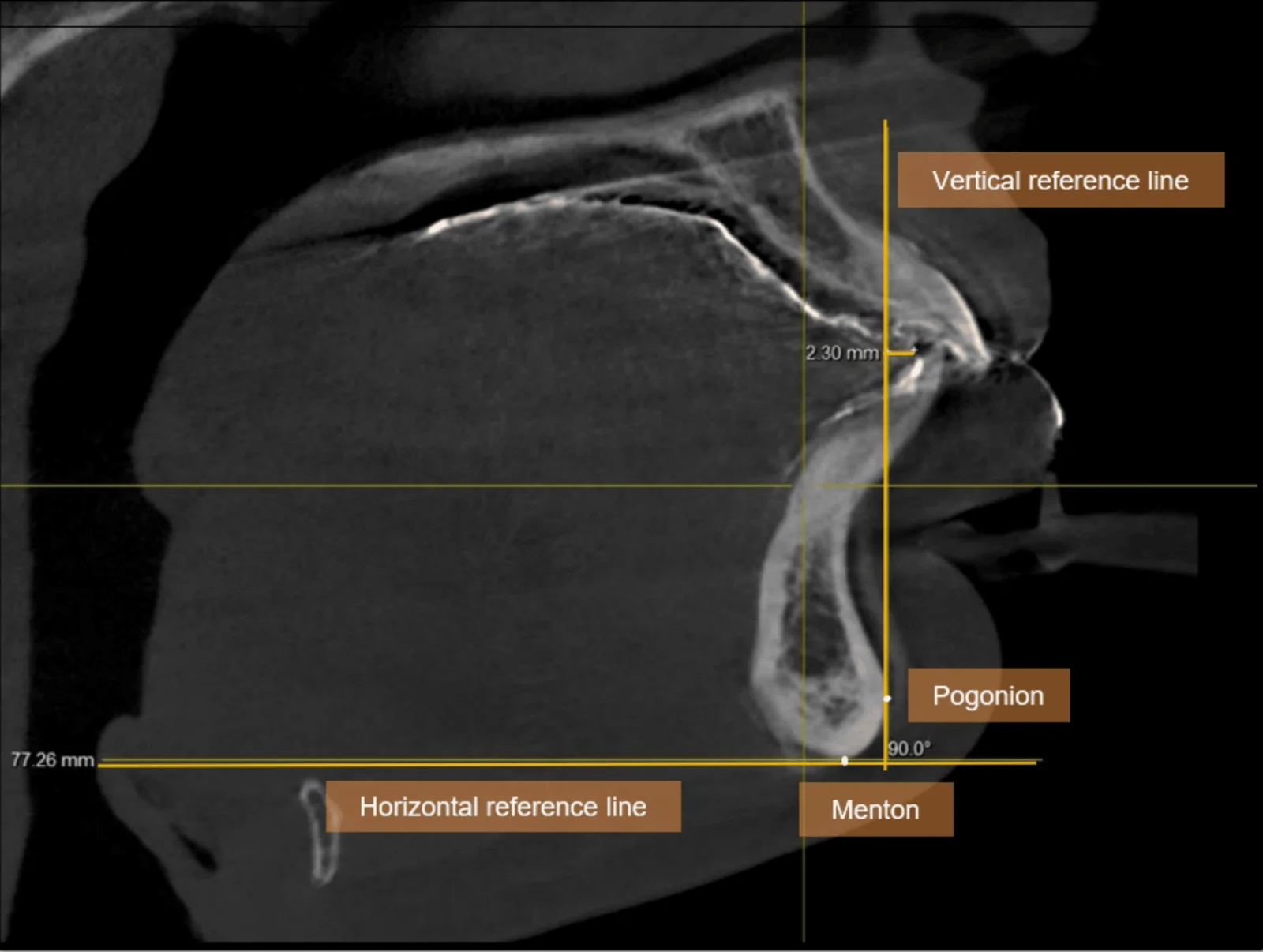
Assessment of tongue tip position on CBCT This figure shows the assessment of tongue tip position by drawing the horizontal reference line passing through the Menton point, then a perpendicular line is constructed through the Pogonion point, which served as a vertical reference line. Tongue tip position is measured from the vertical reference line. CBCT - cone beam computed tomography

For assessment of tongue tip position, a horizontal reference line passing through the menton point was drawn on the CBCT scan and was used as the baseline. A perpendicular line constructed through the Pogonion point served as the vertical reference line. The sagittal position of the tongue tip was determined by measuring the horizontal distance from the tongue tip to this vertical reference line (Figure 4).

**Figure 5:**
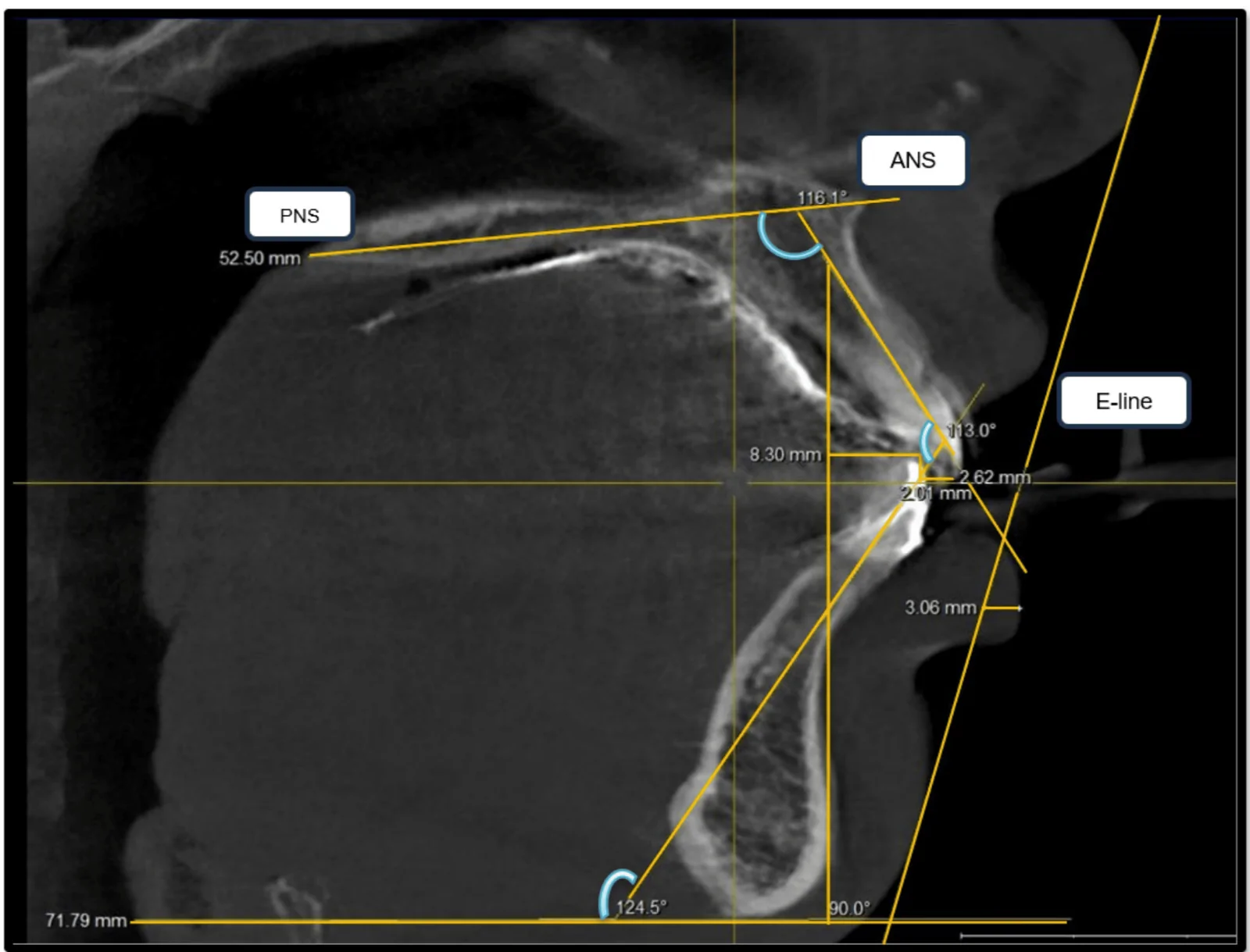
Dento-alveolar angular and linear measurements on CBCT The figure shows the angular measurements measured on the CBCT, such as U1-PP, L1-MP, and interincisal angle, as well as the linear measurements such as tongue tip position, overjet, overbite, and lower lip retraction. ANS - anterior nasal spine; CBCT - cone beam computed tomography; E-line - esthetic line; L1-MP - inclination of lower central incisor to mandibular plane (horizontal reference plane); PNS - posterior nasal spine; U1-PP - inclination of upper central incisor to palatal plane (anterior nasal spine to posterior nasal spine)

Upper incisor inclination was measured as the angle between the long axis of the maxillary central incisor and the palatal plane (anterior nasal spine-posterior nasal spine). Lower incisor inclination was measured as the angle between the long axis of the mandibular incisor and the horizontal reference line. The interincisal angle was measured between the long axes of the maxillary and mandibular central incisors. Overjet was measured as the horizontal distance between the most proclined maxillary incisor and the corresponding mandibular incisor, while overbite was measured as the vertical overlap between the maxillary and mandibular incisors. Lower lip retraction was assessed using the Esthetic line (E-line), extending from the pronasale to the soft tissue pogonion, by measuring the horizontal distance from the most prominent point of the lower lip to the E-line (Figure 5) [13].

The discomfort associated with the appliances was evaluated using a visual analog scale (VAS) ranging from 0 to 10. Parameters including general discomfort, eating difficulty, speech difficulty, tongue irritation, and appliance appearance were assessed on the first day, after one week, and after one month of appliance placement. A score of 0 represented minimal discomfort, whereas a score of 10 represented maximum discomfort. The questionnaires were self-administered after explaining the scoring system to all subjects (Appendices) [10].

Statistical analysis

The obtained data were organized, tabulated, and analyzed using Statistical Package for the Social Sciences (SPSS) software (IBM SPSS Statistics for Windows, IBM Corp., Version 26.0, Armonk, NY). Data normality was assessed using the Shapiro-Wilk test. Mean and standard deviation were calculated for all numerical variables. Intragroup comparisons were performed using the paired t-test for normally distributed data and the Wilcoxon signed-rank test for non-normally distributed data. Intergroup comparisons were conducted using the independent t-test for normally distributed variables and the Mann-Whitney U test for non-normally distributed variables. VAS scores were compared between groups using the Mann-Whitney U test. Baseline age distribution was compared using the independent t-test, while sex distribution was assessed using the chi-square test. A p-value of ≤0.05 was considered statistically significant. The reliability of measurements was examined by re-measuring the measurements on the CBCT scan within a one-week interval. The intraclass correlation coefficient (ICC) was used to evaluate measurement reliability.

## Results

Table 1 displays the demographic data, including the distribution of enrolled subjects' ages and genders in each group. The distribution of enrolled subjects' ages and genders in each group showed no statistically significant difference.

**Table 1:** Subjects' demographic distribution t - independent t-test; χ² - chi-square test Statistical significance was set at p ≤ 0.05.

Demographic Characteristics	Tongue Crib (n = 20)	Tongue Tamers (n = 20)	Statistic	p-value
Age (years), Mean ± SD	19.80 ± 3.44	20.40 ± 4.23	t = 1.261	0.651
Range	14-26	15-30	-	-
Sex, n (%)			χ² = 0.44	0.507
Males	8 (40%)	6 (30%)	-	-
Females	12 (60%)	14 (70%)	-	-

Table 2 shows that the two groups were comparable at baseline for most variables, including tongue tip position, U1-PP, interincisal angle, overjet, overbite, and lower lip retraction (p > 0.05). A significant baseline difference was observed only for L1-MP, where the tongue crib group had a higher mean value than the tongue tamers group (p = 0.014).

**Table 2:** Baseline (pretreatment) comparison ^#^Independent t-test. *Significant difference at p ≤ 0.05. U1-PP indicates inclination of the upper central incisor to the palatal plane (anterior nasal spine to posterior nasal spine); L1-MP indicates inclination of the lower central incisor to the mandibular plane (horizontal reference plane).

Parameter	Tongue Crib (Mean ± SD)	Tongue Tamers (Mean ± SD)	Statistic	p-value
Tongue Tip	-6.02 ± 4.03	-4.06 ± 3.35	-1.672^#^	0.103
U1-PP	120.36 ± 5.38	120.35 ± 7.27	0.007^#^	0.994
L1-MP	126.46 ± 3.06	123.38 ± 3.88	-2.435^#^	0.014*
Interincisal Angle	107.41 ± 5.45	107.28 ± 4.76	-0.609^#^	0.547
Overjet	3.89 ± 2.00	3.29 ± 1.10	1.185^#^	0.243
Overbite	1.58 ± 0.90	1.70 ± 0.67	-0.068^#^	0.947
Lower Lip Retraction	2.57 ± 1.38	2.20 ± 1.67	-0.975^#^	0.341

Table 3 demonstrates that the tongue crib group exhibited statistically significant improvements in all evaluated parameters following treatment. Significant changes were observed in tongue tip position, U1-PP, L1-MP, interincisal angle, overbite, and lower lip retraction (p < 0.001). A significant reduction in overjet was also noted (p = 0.001). These findings indicate that the tongue crib appliance was effective in improving tongue posture and producing favorable dentoalveolar and soft tissue changes.

**Table 3:** Comparison between pre- and post-intervention parameters in tongue crib group ^#^Paired t-test. ^$^Wilcoxon signed-rank test. *Significant difference at p ≤ 0.05. U1-PP indicates inclination of the upper central incisor to the palatal plane (anterior nasal spine to posterior nasal spine); L1-MP indicates inclination of the lower central incisor to the mandibular plane (horizontal reference plane).

Parameter	Pre (Mean ± SD)	Post (Mean ± SD)	Statistic	p-value
Tongue Tip	-6.02 ± 4.03	0.28 ± 4.60	-10.789^#^	<0.001*
U1-PP	120.36 ± 5.38	115.09 ± 6.05	10.051^#^	<0.001*
L1-MP	126.46 ± 3.06	121.56 ± 2.86	-3.921^$^	<0.001*
Interincisal Angle	107.41 ± 5.45	115.14 ± 6.66	-3.921^$^	<0.001*
Overjet	3.89 ± 2.00	2.43 ± 1.27	3.803^#^	0.001*
Overbite	1.58 ± 0.90	2.83 ± 0.72	-6.003^#^	<0.001*
Lower Lip Retraction	2.57 ± 1.38	1.45 ± 1.16	4.723^#^	<0.001*

Table 4 demonstrates that the tongue tamers group exhibited statistically significant improvements in all evaluated parameters following treatment. Significant changes were observed in tongue tip position, U1-PP, L1-MP, interincisal angle, overjet, and lower lip retraction (p < 0.001), except for significant overbite improvement (p = 0.004). These findings indicate that the tongue tamers appliance was also effective in improving tongue posture and producing favorable dentoalveolar and soft tissue changes.

**Table 4:** Comparison between pre- and post-intervention parameters in tongue tamers group ^#^Paired t-test. ^$^Wilcoxon signed-rank test. *Significant difference at p ≤ 0.05. U1-PP indicates inclination of the upper central incisor to the palatal plane (anterior nasal spine to posterior nasal spine); L1-MP indicates inclination of the lower central incisor to the mandibular plane (horizontal reference plane).

Parameter	Pre (Mean ± SD)	Post (Mean ± SD)	Statistic	p-value
Tongue Tip	-4.06 ± 3.35	0.14 ± 3.09	-8.719^#^	<0.001*
U1-PP	120.35 ± 7.27	115.05 ± 6.44	6.619^#^	<0.001*
L1-MP	123.38 ± 3.88	119.87 ± 4.03	8.378^#^	<0.001*
Interincisal Angle	107.28 ± 4.76	114.91 ± 6.24	-8.190^#^	<0.001*
Overjet	3.29 ± 1.10	1.59 ± 0.72	7.372^#^	<0.001*
Overbite	1.70 ± 0.67	2.30 ± 0.82	-3.282^#^	0.004
Lower Lip Retraction	2.20 ± 1.67	1.49 ± 1.57	-3.340^$^	0.001*

Table 5 presents the intergroup comparison of treatment changes between the tongue crib and tongue tamers group. The tongue crib group demonstrated significantly greater tongue tip retraction (p = 0.020) and overbite improvement (p = 0.023), whereas the tongue tamers group exhibited significantly greater overjet reduction (p = 0.002). No statistically significant differences were observed between the groups with respect to U1-PP, L1-MP, interincisal angle, or lower lip retraction (p > 0.05).

**Table 5:** Intergroup comparison of mean change between tongue crib and tongue tamers group (pre-post) ^#^Independent t-test. ^$^Mann-Whitney test. *Significant difference at p ≤ 0.05. U1-PP indicates inclination of the upper central incisor to the palatal plane (anterior nasal spine to posterior nasal spine); L1-MP indicates inclination of the lower central incisor to the mandibular plane (horizontal reference plane).

Parameter	Tongue crib	Tongue Tamers	Statistic	p-value
Tongue Tip Retraction	-6.30 ± 2.27	-4.20 ± 2.16	-2.347^#^	0.020*
U1-PP	5.27 ± 2.35	5.30 ± 3.47	0.192^#^	0.849
L1-MP	4.90 ± 1.18	3.51 ± 1.86	-1.583^$^	0.114
Interincisal Angle	-7.74 ± 4.36	-7.63 ± 3.99	-0.244^$^	0.82
Overjet	1.46 ± 1.29	1.70± 0.88	3.312^#^	0.002*
Overbite	-1.25 ± 0.93	-0.60 ± 0.81	-2.36^#^	0.023*
Lower Lip Retraction	1.12 ± 1.06	0.71 ± 0.72	-0.893^$^	0.383

There was also strong intra-examiner agreement with ICC values ranging from 0.948 to 0.989, indicating a high level of intra-observer reliability and consistency in the recorded measurements.

Table 6 shows the patient perception using VAS scores, in which the tongue crib group showed statistically significantly higher general discomfort, speech difficulty, eating difficulty, tongue irritation, and esthetic concerns in comparison to tongue tamers. 

**Table 6:** Comparison of patient perception *Significant difference at p ≤ 0.05.

Parameter	Time Interval	Tongue Crib	Tongue Tamers	z-value	p-value
General Discomfort	1st day	8.00 ± 2.22	5.60 ± 2.11	-3.013	0.002*
	After 1 week	6.20 ± 2.04	2.75 ± 2.02	-4.044	<0.001*
	After 1 month	3.70 ± 2.11	0.90 ± 1.62	-4.204	<0.001*
Speech	1st day	8.60 ± 2.14	4.05 ± 2.56	-4.413	<0.001*
	After 1 week	6.90 ± 2.02	2.60 ± 2.23	-4.363	<0.001*
	After 1 month	4.80 ± 2.17	0.95 ± 1.19	-4.668	<0.001*
Eating	1st day	8.05 ± 2.72	3.50 ± 2.46	-4.666	<0.001*
	After 1 week	6.55 ± 2.63	1.95 ± 1.76	-4.862	<0.001*
	After 1 month	4.60 ± 2.84	0.75 ± 1.29	-4.635	<0.001*
Tongue Irritation	1st day	7.90 ± 2.20	4.45 ± 2.70	-3.665	<0.001*
	After 1 week	5.70 ± 2.13	2.10 ± 1.52	-4.481	<0.001*
	After 1 month	3.45 ± 2.31	0.75 ± 0.79	-4.418	<0.001*
Appearance	1st day	6.90 ± 1.77	0.65 ± 1.42	-5.469	<0.001*
	After 1 week	5.00 ± 1.69	0.40 ± 0.97	-4.36	<0.001*
	After 1 month	2.65 ± 1.81	0.05 ± 0.22	-5.232	<0.001*

## Discussion

Tongue thrusting is considered an important etiological factor in the development and persistence of malocclusion due to the continuous abnormal tongue posture and altered swallowing pattern. According to Proffit’s equilibrium theory, the duration of force exerted by the tongue is more important than its magnitude, and prolonged abnormal tongue posture can result in proclination of incisors, increased overjet, anterior open bite, and altered facial musculature [14].

In the present study, tongue thrust swallowing was diagnosed clinically by asking the subjects to swallow their saliva three times during the same visit, and the diagnosis was confirmed independently by two clinicians to improve reliability and reduce examiner bias [15]. The use of CBCT imaging enabled 3D evaluation of tongue posture and associated dentoskeletal structures without superimposition errors. In addition, coating the dorsal surface of the tongue with barium sulphate enhanced its visualization, thereby allowing more precise assessment of tongue posture changes [16].

The results of the present study demonstrated statistically significant improvement in tongue tip position in both the tongue crib and tongue tamers groups. Tongue tip retraction was significantly greater in the tongue crib group (-6.30 ± 2.27 mm) compared to the tongue tamers group (-4.20 ± 2.16 mm), indicating superior efficacy of the tongue crib in repositioning the tongue posteriorly. The increased effectiveness of the tongue crib can be attributed to the mechanical design of the appliance, which acts as a physical barrier and directly restricts the anterior tongue thrusting during swallowing and speech. The appliance disrupts the abnormal muscular pattern and promotes adaptation to a more posterior tongue posture. Similar results have been reported in previous studies assessing the effectiveness of palatal crib therapy in correction of tongue thrusting and anterior open bite. Rossato et al. [17] reported significant dentoalveolar correction with fixed palatal crib, indicating its greater effect on incisor positioning.

Although tongue tamers also resulted in significant improvement in tongue posture, their effectiveness was comparatively lower than tongue crib. This may be due to gradual adaptation of the tongue to the appliance over time, thereby reducing its proprioceptive stimulation effect. Additionally, high debonding rates (86.67%) were associated with tongue tamers, which might have compromised the treatment efficiency and patient compliance. Tongue tamers primarily function by proprioceptive feedback rather than direct mechanical restriction, which may explain the comparatively lesser amount of tongue tip retraction observed in the present study [18]. 

Upper incisor inclination (U1-PP) decreased significantly in both groups, indicating retroclination of maxillary incisors following correction of tongue thrusting habit. The reduction was 5.27 ± 2.35° in the tongue crib group and 5.30 ± 3.47° in the tongue tamers group, with no statistically significant intergroup difference. This reduction may be explained by elimination of excessive anterior tongue pressure on the maxillary incisors, thereby allowing normal lip musculature to establish equilibrium. These findings are in accordance with the study conducted by Alawy et al. [19], who also observed statistically insignificant changes in the upper incisor inclination and observed that the overbite was corrected by extrusion alone, since there were no inclination changes. Similar findings were reported by Leite et al. [18], who found minor but favorable changes in upper incisor inclination following palatal crib therapy after 12 months of follow-up.

Lower incisor inclination (L1-MP) showed a significant reduction of 4.90 ± 1.18° in the tongue crib group and 3.51 ± 1.86° in the tongue tamers group, indicating favorable retroclination and repositioning of the mandibular incisors. However, the intergroup comparison revealed no statistically significant difference, suggesting that both appliances were effective in reducing lower incisor inclination. The retroclination of the mandibular incisors may be attributed to the elimination of abnormal tongue pressure and the subsequent unopposed action of the lower lip musculature. These findings are consistent with those reported by Shams et al. [9], Rossato et al. [17], and Leite et al. [18], who also observed significant retroclination of the lower incisors following treatment with a fixed palatal crib. In contrast, previous studies evaluating tongue tamers reported minimal changes in L1-MP, raising concerns regarding their ability to effectively control lower incisor inclination. The present study, however, demonstrated significant reductions in L1-MP within the tongue tamers group, suggesting that this appliance may also contribute to favorable mandibular incisor repositioning.

The interincisal angle significantly increased by -7.74 ± 4.36° and -7.63 ± 3.99° in the tongue crib and tongue tamers group, respectively, reflecting improved axial inclination of incisors in both groups. The increase observed in the tongue crib group is comparable with findings reported by Shams et al. [9] and Rossato et al. [17], who demonstrated improvement in incisor relationship after palatal crib therapy. The increase in interincisal angle reflects improvement in anterior incisor inclination resulting from correction of abnormal tongue posture and elimination of forward tongue pressure.

Significant improvements in both overbite and overjet were observed following treatment in the tongue crib and tongue tamers groups. Intergroup comparison demonstrated that the tongue crib group achieved significantly greater improvement in overbite than the tongue tamers group. This improvement may be attributed to the elimination of the tongue-thrusting habit, which permits normal eruption of the anterior teeth and facilitates the correction of anterior open bite. These findings are in agreement with those of Shams et al. [9], Rossato et al. [17], and Alawy et al. [19], who reported successful correction of anterior open bite following treatment with tongue crib appliance.

Conversely, the tongue tamers group exhibited a significantly greater reduction in overjet compared with the tongue crib group. This finding may be related to the correction of the tongue thrusting habit, which contributed to the retroclination of both the maxillary and mandibular incisors. Additionally, the comparatively lower pretreatment lower incisor inclination observed in the tongue tamers group may have influenced the magnitude of overjet reduction achieved. Therefore, the greater improvement in overjet observed in this group should be interpreted with consideration of the baseline differences between the groups, in addition to the treatment effects of the appliance itself.

The lower lip also got retracted significantly in both groups, suggesting favorable soft tissue adaptation following correction of incisor proclination and tongue posture. The reduction in protrusion of the lower lip relative to the esthetic line (E-line) may be associated with improved muscular balance and retroclination of incisors. However, no statistically significant difference was observed between the two groups, indicating that both appliances produced comparable soft tissue improvements.

VAS was used to assess patient comfort and perception. The results demonstrated significantly greater discomfort, speech difficulty, eating difficulty, tongue irritation, and esthetic concerns in the tongue crib group compared to tongue tamers on the first day, after one week, and one month following the appliance placement. These findings may be explained by the bulky design and mechanical obstruction produced by the tongue crib, which may interfere with tongue movements, speech articulation, and oral hygiene. Previous studies have similarly reported tongue irritation, speech difficulties, and reduced patient comfort with fixed palatal crib [10]. In contrast, tongue tamers are smaller, less invasive, and more esthetic appliances, thereby improving patient adaptability and comfort.

Clinical implications

In clinical practice, the choice between a tongue crib and tongue tamers should achieve a balance between effectiveness and patient compliance and comfort. The tongue crib is an effective appliance when immediate changes in tongue position are needed or when there is severe malocclusion. It can be used in combination with myofunctional exercises to enhance neuromuscular adaptation. On the other hand, tongue tamers may be considered the ideal choice where comfort and esthetics are important or in less severe cases, but they can also lead to patient adaptation and debonding.

The limitation of this study is that it is a small-scale, single-center clinical trial with a relatively short follow-up period of six months. Long-term studies with larger sample sizes are required to evaluate the stability of treatment outcomes and relapse tendency following appliance removal. Additionally, patient compliance and adaptation to appliances may influence the final treatment outcome and should be further investigated. Involuntary swallowing movements due to nervousness or anxiety may temporarily elevate or retract the tongue, affecting the accuracy of tongue position assessment.

## Conclusions

Both tongue crib and tongue tamers are effective in the management of tongue thrusting habit and produce favorable dentoalveolar changes. Although both appliances can be successfully used for habit interception, the tongue crib may be preferred in cases requiring greater correction, such as in anterior open bite cases. However, its use is associated with increased patient discomfort, speech and eating difficulties, tongue irritation, and esthetic concerns. In contrast, tongue tamers may represent a more patient-friendly alternative, offering better comfort and acceptance despite comparatively lesser treatment effects and a higher incidence of debonding.

The use of CBCT enabled a comprehensive 3D assessment of treatment-related changes. Unlike conventional two-dimensional imaging, CBCT provided precise visualization and measurement of tongue tip position, dentoalveolar structures, and soft tissue adaptations, thereby enhancing the accuracy and reliability of outcome evaluation. The findings of this study suggest that both appliances are viable treatment options for the correction of tongue thrusting habit, with the choice of appliance guided by the desired treatment outcomes and individual patient requirements.

## Appendices

**Table 7:** Visual analog scale questionnaires Credits: Dr. Simmi Arora, Dr. Pradeep Raghav, Dr. Ruchi Saini, Dr. Anamika Sharma, Dr. Manvi Agarwal Participants were asked to indicate their perception regarding the tongue crib and tongue tamers appliance by marking a point on a 10-cm VAS for each question. A score of 0 represented the absence of discomfort or difficulty, whereas a score of 10 represented the maximum possible discomfort or difficulty. Assessments were recorded at three time points: on the first day, after one week, and after one month of appliance placement.

Parameter	Question Asked to the Patient	Scale Interpretation
General Discomfort	Mark your level of overall discomfort caused by the appliance.	0 = no discomfort; 10 = worst possible discomfort
Speech Difficulty	Mark how difficult it is for you to speak with the appliance.	0 = no difficulty; 10 = extremely difficult
Eating Difficulty	Mark how difficult it is for you to eat with the appliance.	0 = no difficulty; 10 = extremely difficult
Tongue Irritation	Mark the level of irritation or discomfort experienced by your tongue.	0 = no irritation; 10 = worst possible irritation
Appearance	Mark your level of concern regarding the appearance of the appliance.	0 = no concern; 10 = maximum concern
